# In Silico Modeling of Stress Distribution in the Diseased Ankle Joint

**DOI:** 10.3390/jcm13185453

**Published:** 2024-09-13

**Authors:** Jacek Lorkowski, Miroslaw W. Mrzyglod, Mieczyslaw Pokorski

**Affiliations:** 1 Faculty of Physics and Engineering Sciences, University of Surrey, Guildford GU2 7XH, UK; jacek.lorkowski@gmail.com; 2 Department of Management and Accounting, SGH Warsaw School of Economics, 02-554 Warsaw, Poland; 3 Department of Mechanical and Automobile Engineering, Technological University of the Shannon, Moylish Campus, Moylish Park, V94 EC5T Limerick, Ireland; miroslaw.mrzyglod@tus.ie; 4 Institute of Health Sciences, Opole University, 45-040 Opole, Poland

**Keywords:** age, bone stress, body weight, ankle joint, in silico analysis, joint degeneration

## Abstract

**Background/Objectives:** Osteoarthritis is a feature of the aging process. Here, we adopted in silico 2D finite element modeling (FEM) for the simulation of diseased ankle joints. We delved into the influence of body weight intensity on the stress distribution caused by subchondral cysts imitating degenerative age-related arthritic changes. **Methods:** FEM was performed using virtually generated pictorial schemes of the ankle joint skeletal contour. It included a constellation of scenarios with solitary or multiple cysts, or the lack thereof, located centrally, peripherally, or both in the talus and tibia at increased fixed levels of body weight. **Results:** The modeling showed that the highest stress was in the presence of a solitary central cyst in the talus and two centrally located cysts in the talus and the tibia, with the averaged values of 1.81 ± 0.52 MPa and 1.92 ± 0.55 MPa, respectively; there was a significant increase compared with the 1.24 ± 0.35 MPa in the control condition without cysts. An increase in body weight consistently increased the strain on the ankle joint. In contrast, peripherally located cysts failed to affect the stress distribution significantly. **Conclusions:** We conclude that subchondral central cysts substantially enhance the stress exerted on the ankle joint and its vicinity with body weight dependence. FEM’s ability to predict the location and magnitude of subchondral stress changes when confirmed in clinical trials might help to optimize the management of age-related degenerative joint changes.

## 1. Introduction

The importance of the musculoskeletal system function for the population’s health and particularly the aging process is best demonstrated by the WHO-driven past program of The Bone and Joint Decade 2000–2010 and the current The Decade of Healthy Aging 2021–2030, which includes the prevention and treatment of injuries and degenerative changes in the elderly [1,2]. Degeneration typically affects the spine, knee, and hip joints. Osteoarthritis of the ankle joint, albeit about 10 times rarer, affects at least 1% of the adult population, with increasing frequency in advanced age [3]. Moreover, obesity is strongly associated with higher fall rates in the elderly; the authors of [4] have reported a 25% to 31% higher risk of falls. It is clinically and socioeconomically burdening, causing pain, dysfunction, and limited mobility. It appears that the stability of the ankle joint system is key for musculoskeletal health [5]. Further, the relationship between idiopathic osteoarthritis of the ankle joint interacts with post-traumatic osteoarthritis, the frequency of either being potentiated in advanced age [6,7]. The average time gap between the occurrence of injury to the development of degenerative ankle disease amounts to 21 years, with a range of 1–52 years [8].

Computerized in silico finite element modeling (FEM) is a method proficiently used in orthopedics to delve into bone and joint stress distribution in conditions simulating real-life clinical experience [9,10]. The method enhances the comprehension and retention of molecular bone arrangements forming bone stability due to the cross-alignment of normal stress under axial loading, like the sheer body weight, twisting stress, as in a joint shaft, and bending stress distribution. FEM is also increasingly suggested as a method to guide management and interventions in the realm of ankle joint orthopedics. The method has been successfully used for the ankle joint modeling of stress distribution in various disorders that predispose patients to the onset of osteoarthritic changes, like varus deformities developing with age or post-trauma due to increased joint weight bearing causing the elevation of cartilage contact pressure [5,11]. FEM is also used to simulate the effects of local fractures on ankle biomechanics and to predict patterns of tibiotalar pressure-induced displacements and osteotomy management [12,13].

The bone stress distribution is primarily affected by degenerative age-related conditions, the most common of which are subchondral cysts in the ankle joint bone system. Nonetheless, there is a paucity of FEM studies on the pathologic properties of cysts. A lonely, relevant study accessible to us in the medical literature was provided by Talbott et al. [14]. Using magnetic resonance images, that clinical study evaluated naturally developed subchondral cysts in ankle joint arthropathy in hemophiliacs. Although the contact pressure was redistributed, a distinct relation to the location, shape, volume, and number of cysts could not be evidenced. In the present study, we performed FEM on virtual pictures of ankle joint bone contours, which allowed us to fill in the fixed outlines, volume, shape, and in-bone location of cysts, and, by doing so, to circumvent the cysts’ natural multifariousness. We showcase examples of stress distribution caused by central and peripheral subchondral cysts in the ankle joint, considering the impact of body weight bearing.

## 2. Material and Methods

The in silico 2D FEM consisted of an assessment of the stress distribution occurring at the interface of the ankle and adjacent foot joints, particularly concerning the subchondral layer of the ankle joint in the vicinity of subchondral cysts. The study was a virtual meta-analysis that did not involve any human subjects, patients, or data. The modeling was based on virtually generated pictorial schemes of the ankle joint skeletal contour in the frontal and sagittal projections. The contours were monochromatic and devoid of internal bone content. The torsion aspect was not assessed. The sagittal projection passed through the center of the talus’s trochlea. The frontal projection included the plane in which both the frontal and fibula bones were present. Thus, it was taken posteriorly to the plane passing through the center of the talus’s trochlea.

### 2.1. Modeling Data

The picture resolution we used for this modeling was 250 × 250 pixels. The FEM modeling started from the assignment of 256 material attributes consisting of bitmap shades in the picture grayscale using the CT2FEM v1.0 software (Mrzyglod, Cracow University of Technology, Cracow, Poland). The bone cross-sectional surface was modeled using eight-node solid linear finite elements of 0.5 mm in size, grouped according to the individual material properties considered. The mesh was not optimized given the assumption of fast modeling. The model’s boundary conditions consisted of restraining at its base and loading with distributed force according to gravity at its top. The boundaries corresponded to the areas normally formed by healthy bone tissue, the articular cartilages of the talocrural, talocalcaneal, and talonavicular joints, ligaments, and small fluid spaces. Regarding the degenerative changes, fluid-filled cysts were considered. The cysts were superimposed on the bone contour scheme by hand in central and peripheral locations. Their size grossly averaged that of in vivo degenerative conditions [15].

**Figure 1:**
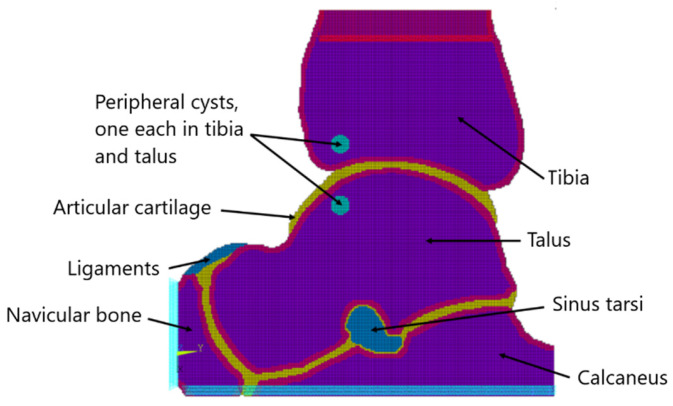
Schematic representation of the ankle joint system with two peripheral cysts and boundary conditions applied to the model.

The numerical model consisted of 4039 finite elements of two types (Type 1: SOLID185; Type 2: MESH200). A scheme of the FEM model is shown in Figure 1 together with the boundary conditions applied. The visualization of the restraint in the z direction was turned off so as not to obscure the picture. Type 2 elements could be parametrically assigned to any area of the model and adapted for model modification. The pressure loads for various model scenarios were defined in the Ansys Parametric Design Language (APDL), a structured scripting language run in batch mode that provided results in the form of contour maps of the von Mises equivalent stresses. Material properties were homogenized throughout the modeling. Homogenization included trabecular and cortical bones whose structures have innate real-life focal differences, which underscores the modeling impersonality.

Seven modeling scenarios were considered with various configurations of centrally and peripherally located cysts in the talus and tibia, as enumerated in the qualitative results below. The control reference was a scenario with no cyst and a standard body weight of 70 kg. Five intervention scenarios consisted of sequential body weight load increases to 90 kg, 110 kg, 130 kg, 150 kg, and 170 kg (Table 1). A simplified, uniform description of tissue properties was adopted for the numerical research. For the cortical bone, Young’s modulus was E = 20 GPa and Poisson’s number was ν = 0.3, and for the trabecular bone, E = 10 GPa and ν = 0.3 [16]. Accordingly, E = 1 MPa and ν = 0.3 were assumed for the articular cartilage, E = 1 kPa and ν = 0.3 for the soft tissues, E = 10 MPa and ν = 0.3 for the ligaments, E = 1 kPa and ν = 0.3 for the fluid spaces, and E = 1 kPa and ν = 0.3 for the medullary cavity. In each model, a static analysis was performed for the adopted load pattern, in which the load was distributed evenly over the cross-section. The processing was supported by removing all degrees of freedom in the nodes at the bottom edge of the model. For a 70 kg patient, the FEM was loaded with a force corresponding to an average load of 0.248 N/mm^2^, the vector of which was consistent with the limb axis. The respective loading forces in the models with the sequential increases in body weight were 0.318, 0.389, 0.460, 0.530, and 0.601 N/mm^2^. The spatial stress distribution was taken as a measure of the effort of the bone or fixing material, and not as a single tension deviation before a fracture or deflection, according to the Huber–Mises–Hecky hypothesis [17,18].

### 2.2. Statistical Elaboration

Statistical data elaboration consisted of the non-parametric Friedman ANOVA test for differences in the ankle joint stress among the seven cyst modeling scenarios, including their number and location, each at six fixed body weight loads, presented as columns vs. rows in Table 1. The Wilcoxon matched-pairs test was used for the pairwise scenario comparisons. Additionally, the Pearson correlation coefficient was used to assess the effects of increased body weight on the ankle joint stress in each modeling scenario. Statistically significant differences were assumed at α = 0.05.

## 3. Results

### 3.1. Qualitative FEM Data

**Figure 2:**
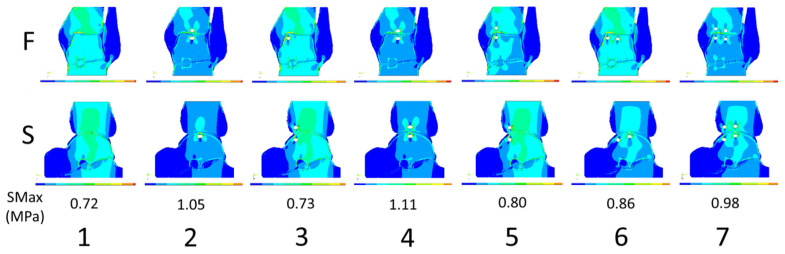
Stress distribution in the in silico 2D finite element modeling (FEM) of ankle joint degeneration consisting of subchondral cysts in a standard patient weighing 70 kg illustrated in the frontal (F—upper row) and sagittal (S—lower row) planes. The columns of modeling scenarios 1 to 7 correspond to the sequence listed at the top of the Qualitative FEM Data subheading above. Column 1, which does not contain diseased changes, is a reference for comparisons; SMax, stress maximum.

The 2D illustrations of the in silico FEM technique shaping the ankle joint content are demonstrated in the sequential panels of Figure 2. We performed a constellation of modeling scenarios as follows:

1. No cyst.
2. Solitary central cyst in the talus.
3. Solitary peripheral cyst in the talus.
4. Two central cysts, one of each in the talus and the tibia.
5. Two peripheral cysts, one of each in the talus and the tibia.
6. Two cysts, a central and a peripheral in the talus.
7. Four cysts, a pair of central and peripheral each in the talus and the tibia.

In the absence of cysts, the highest stress was found on the upper articular surface just medial to the central shin axis in the frontal plane (Figure 2(1F)) and centrally in the limb axis in the sagittal plane (Figure 2(1S)). A single central cyst induced the highest stress around its vicinity (Figure 2(2F,S)). A single peripheral cyst also induced a rise in stress around itself, but this did not significantly disturb the high stress in the limb axis (Figure 2(3F,S)). Two central cysts, one in the talus and the other in the tibia, induced the highest stress around themselves. The area of increased stress is shaped like a butterfly with cysts at the central axis (Figure 2(4F,S)). Two peripheral cysts, one in the talus and the other in the tibia, induced stress around themselves grossly like that in the absence of cysts, except for a higher stress area extending from the side of the cyst in the talus in the frontal plane (Figure 2(5F,S)). Two cysts in the talus induced higher stress around and lower beneath themselves (Figure 2(6F,S)). In the frontal plane, the highest stress domain was consistent with the limb axis, with greater stress in the tibia than the talus. Four cysts triggered the highest stress centrally in the tibia and the upper part of the talus in the frontal plane. In the sagittal plane, the highest stress appeared close to the long limb axis reaching the heel bone level (Figure 2(7F,S)).

### 3.2. Quantitative FEM Data

Modeling in the frontal and sagittal planes yielded closely overlapping results. Therefore, the results from the two combined planes were averaged for further presentation. Table 1 shows the von Mises stress values occurring with the configuration and location of the degenerative subchondral cysts outlined above, with each increasing body weight load starting from the standard control of 70 kg.

The ANOVA Friedman test indicated that there was a significant difference in the mean stress level between the modeling scenarios of the ankle joint degenerative cyst-emulating stress and the means of 1.24 MPa for S1, 1.81 MPa for S2, 1.26 MPa for S3, 1.92 MPa for S4, 1.32 MPa for S5, 1.48 MPa for S6, and 1.70 MPa for S7 (*p* < 0.0001). The Wilcoxon signed-rank test indicated that the mean stress levels of the following pairs were significantly different: x1–x2, x1–x3, x1–x4, x1–x6, x1–x7, x2–x3, x2–x4, x1–x5, x2–x6, x2–x7, x3–x4, x3–x6, x3–x7, x4–x5, x4–x6, x4–x7, x4–x7, x5–x6, and x5–x7 (all *p* < 0.03). Thus, stress levels were significantly higher in the scenarios with cysts than in the reference scenario (S1) devoid of cysts, except for S5 containing two peripheral cysts, in which the stress did not significantly exceed the reference. Generally, central cysts induced higher stress than peripheral ones (S2 vs. S3 and S4 vs. S5).

**Figure 3:**
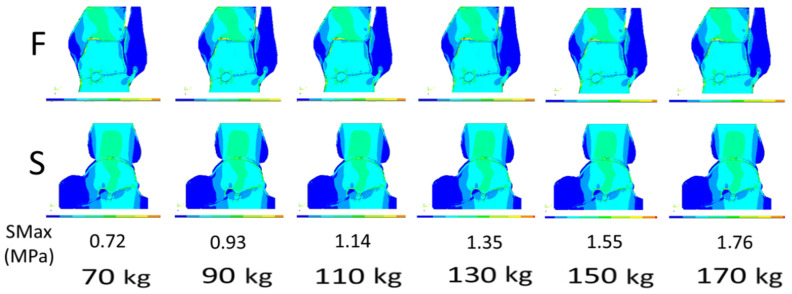
Strain distribution in the in silico 2D finite element modeling (FEM) in the ankle joint with increasing patient body weight. All illustrations present a normal joint without any diseased changes. A standard patient weighing 70 kg was taken as a reference for comparisons. Illustrations are in the frontal (F—upper row) and sagittal (S—lower row) planes; SMax, stress maximum.

The effect of strain exerted on the ankle joint by the body weight was investigated in FEM illustrations presenting sequential increases in weight from 70 kg to 170 kg. A standard patient of 70 kg was taken as a reference for comparisons, all of which addressed the joint condition with no diseased changes. The strain distinctly and invariably increased with the body weight increases (Figure 3). It perfectly positively correlated with body weight. A body weight increase of 1 kg enhanced the strain by 0.01 MPa (scenarios 1, 3, 5, 6, and 7) and 0.02 MPa (scenarios 2 and 4).

## 4. Discussion

The distribution of forces acting on the cartilage and the richly innervated subchondral layer of the ankle joint is influenced by local macro- and microanatomical features. In the present 2D FEM, we investigated the potential influence of subchondral cysts, a frequent diseased or degenerative age-driven occurrence in the joint. The findings showed that the cyst number and bone location mattered. The cysts located centrally along the limb axis, either solitary or occurring simultaneously in the talus and the tibia, appreciably increased the bone stress, whereas it was less so for the peripheral ones. Interestingly, the accompaniment of peripheral cysts significantly lowered the stress exerted by the central cysts alone. This observation may have clinical implications for clinical decision making concerning the filling of central cysts or surgical treatment of peripheral cysts. The intertwined relationship between various cysts is explicable by physical forces. The superposition of cysts in the central axis of the pressure load causes the articular cartilage to behave like thick fluid and “spread” sideways from the biomechanical standpoint. As a result, the fluid’s functional thickness in the central part of the joint becomes relatively smaller, which increases the contact stress [19]. Another plausibility is the impact of structure perforation on the ratio of the effective Young’s modulus. Evenly spaced perforations in a continuous structure reduce Young’s modulus proportionally to the mass loss, i.e., the perforation number. From the point of view of solid mechanics, the material becomes more elastic and bends more under the same load [20]. Additionally, we found that increased body weight, a frequent accompaniment of old age [4], resulted in linearly increasing loading stress on the ankle joint. In the case of cyclic loading, which occurs while standing and walking, microcracks develop in the ankle joint, enhancing degenerative changes and joint damage.

The literature on in silico-modeled cysts in the ankle joint is rare. The only pertinent study for comparison was that by Talbott et al. [14]. Those authors performed FEM to assess the influence of subchondral bone cysts on ankle joint health concerning their shapes, volumes, and depths in hemophilic patients. The study was performed on magnetic resonance image scans. The authors reported a redistribution of cartilage contact pressures. However, a great variety of cyst structural characteristics and localizations in a small number of patients made it difficult to establish distinct relationships. Moreover, the patients suffered from hemarthrosis, a specific kind of arthropathy [21], which might further impede conclusions. That contrasts with the present study based on the impersonal, virtually idealized, and structurally fixed cysts. Such a study design was our purposeful choice. It introduces a bias of detachment from the clinical reality but allows us to study subchondral contact pressure distribution changes in structurally and positionally fixed cysts, circumventing the issue of their natural multifariousness.

Microstructurally, the extracellular matrix of articular cartilage consists mainly of proteoglycans and type II collagen, which supports an almost friction-free joint movement [22]. Structural damage of the subchondral layer disturbs the intra-joint forces, which perpetuates the damage, often resulting in pain symptoms. Such processes take place throughout the entire kinetic joint chain. In the ankle joint, however, the extracellular matrix is denser than, for instance, in the knee joint, with less water and more glycosaminoglycans. During compression, the ankle cartilage has a higher elastic modulus and dynamic stiffness than the knee cartilage. In young people, tensile stress is higher in the femur head than in the talus. Such differences may even out with age; for instance, stress decreases faster in the hip than in the ankle joint [23]. The articular cartilage thickness factors in while comparing the ankle and other large joints. The cartilage is outstandingly thinner in the ankle joint than in the knee or hip joint, at 0.70–1.62 mm vs. 1.5–2.6 mm vs. 3–6 mm, respectively, which conditions the biomechanical differences [24].

The articular cartilage metabolism in the ankle joint has specific characteristics. The cartilage contains fewer collagenase receptors, particularly for metalloproteinase 8 (MMP-8), than, for instance, that in the knee joint. These receptors react with interleukin-1 (IL-1) to inhibit proteoglycan synthesis, which therefore is several times smaller than in the knee joint [25]. The ankle cartilage also is more resistant to degradation. It does not change significantly after exposure to the fibronectin fragment (Fn-f) for a month [26]. The proteoglycan content is reduced by 30–50% in the knee cartilage during a two-week Fn-f exposure. Such differences undoubtedly influence the susceptibility for osteoarthritis across joints.

At the macroanatomical level, the ankle joint has noticeably smaller surface contact between opposing articular surfaces compared to the knee or hip joint. It is a compact joint, and the loading forces normally act to keep its stability [27]. During foot pressing, the forces transmitted by the ankle joint correspond to approximately 4.5–5.5 times the weight of the body. The complex surface geometry of the ankle joint variably affects its contact surfaces. The acting forces depend on the joint’s position in which it is body-weight-loaded [28]. Our modeling scenarios considered the joint compactness in the so-called zero anatomical position in both frontal and sagittal planes. The modeling showed how the presence of subchondral cysts in various locations may affect the ankle joint’s biomechanics under increasing heavy-body-weight loading. However, the FEM analysis we performed left out the adjacent joints, particularly the talocalcaneal joint, which functions in tandem with the ankle joint. The ankle joint is mainly responsible for movements close to the sagittal plane, whereas the talocalcanealnavicular joint is in the frontal plane. This complex forms a kinetic connection between the shin and the foot, which allows for proper standing and walking. Body weight is taken on mainly by the tibial bone. The role of the fibula is to stabilize the ankle joint. It is estimated that approximately 6.4–20.0% of body weight is carried by the fibula and the remainder by the tibia [29]. We introduced such assumptions in the FEM modeling. During dorsiflexion, the articular surface contacts decrease by over 40%, which, interestingly, may explain the frequency of degenerative changes among ballet dancers [30]. During inversion and eversion movements, the contact area also decreases. Computer models presented in the literature have shown that, during inversion and plantar flexion movements when landing on the forefoot, the possibility of torsion injuries significantly increases, which is a predicament of sports activity [31]. Even a temporary disruption of the joint congruence may have a deteriorating influence. A shift of about 1 mm of the upper, medial, or lateral articular surfaces of the talus relative to the distal ends of the tibia and fibula during non-physiological pressure on the ground reduces the contact surface by 42%, which is prone to produce damage to the ligament apparatus [29,32].

The present study had several limitations inherent to modeling designs. Our design had a substantial degree of subjectiveness in incorporating simplifications, material properties, and boundary conditions. Moreover, the study concerned virtualized conditions, where the number of cysts, location, and volume in the ankle bones were fixed. The model was impersonalized and detached from real clinical conditions. However, as mentioned above in the discussion, the choice of this model was a conscious act to circumvent the all-too-often confounding factors linked to the great variability of diseased conditions present in the realm of orthopedics, where there are hardly two pathologies constituted in a like manner. In our opinion, such confounders make it hardly possible to arrive at distinct conclusions on the actual biomechanical behavior of the ankle joint and are no less limiting than an idealized model. The results of the present imaginary modeling, for instance, showing for the first time significantly greater harmful effects on the subchondral stress of central than peripheral cysts or increased body weight, should be counterchecked in clinically oriented trials before reaching potential applicability judgment.

FEM belongs to universal computerized metamodels thought as a surrogate for a complicated and pricey simulation process [33,34]. It can provide a prompt overview of the distribution of forces acting at the interface of tissues such as bone, articular cartilage, ligaments, fluid spaces, and the vicinity, an assessment that can be otherwise hardly available in the case of sudden falls or trauma requiring immediate medical management [12]. The method can be useful in clinical settings, particularly in automated batch processing to predict the location and magnitude of stress changes, and help undertake preventive and therapeutic measures.

## 5. Conclusions

The in silico 2D finite element modeling performed in this study showed that subchondral cysts in the ankle joint, which are frequent old-age-driven degenerative changes, might substantially enhance joint stress. The cysts located centrally in the tibia and the talus along the limb axis exerted a stronger stress-related increase than the peripheral ones. Stress increased linearly with body weight, which points to gravity-force-related joint strain. The increased stress might disturb the kinetics of the ankle joint and foot, resulting in foot and gait instability, tipping over, and falls, with potentially dire health consequences and disability. FEM’s ability to predict the exact location and magnitude of stress changes when confirmed in clinical trials might help undertake preventive or therapeutic actions to optimize the clinical decision-making process.

## Figures and Tables

**Table 1:** Stress distribution in the in silico 2D finite element modeling (FEM) of ankle joint degeneration consisting of subchondral cysts in consecutive modeling scenarios (S) with each increased body weight.

Body Weight (kg)	Modeling Scenario (S) and Stress Expressed in MPa (Megapascals)						
	S1	S2	S3	S4	S5	S6	S7
70	0.72	1.05	0.74	1.12	0.81	0.86	0.99
90	0.93	1.36	0.94	1.44	1.04	1.11	1.27
110	1.14	1.66	1.16	1.76	1.27	1.35	1.55
130	1.35	1.96	1.36	2.08	1.05	1.59	1.82
150	1.55	2.27	1.58	2.40	1.73	1.85	2.15
170	1.76	2.57	1.78	2.72	2.00	2.09	2.40
*X* ± σ	1.24 ± 0.35	1.81 ± 0.52	1.26 ± 0.36	1.92 ± 0.55	1.32 ± 0.42	1.48 ± 0.42	1.70 ± 0.49

Columns S1 to S7 correspond to the modeling scenarios listed at the top of the Qualitative FEM Data subsection. Column S1, which does not contain diseased changes, is a reference for comparisons. Data subheading: *X* ± σ means ± standard deviation.

## Data Availability

The data presented in this study are available on request from the corresponding author.
